# Analysis of microhabitat characteristics at roost sites of Cerulean Warblers

**DOI:** 10.1371/journal.pone.0241501

**Published:** 2020-11-03

**Authors:** Clayton D. Delancey, Kamal Islam

**Affiliations:** Department of Biology, Ball State University, Muncie, Indiana, United States of America; Feroze Gandhi Degree College, INDIA

## Abstract

Little information exists on songbird roosting habits and the types of habitats that songbirds select. To better understand a species’ habitat requirements, all aspects of its biology should be studied. The Cerulean Warbler (*Setophaga cerulea*, Wilson) is a Nearctic-Neotropical migrant that is a species of conservation concern across its range. It is one of the fastest declining species of wood-warbler (Parulidae) in North America. Since 2007, a breeding population of Cerulean Warblers has been monitored in southern Indiana, as part of the Hardwood Ecosystem Experiment. The Hardwood Ecosystem Experiment is a 100 yr project that studies the effects of different forest management practices on plant and animal communities. During the 2017 breeding season, 10 male Cerulean Warblers were tracked to roost locations. Roost sites selected by male Cerulean Warbler were characterized with less basal area, higher canopy cover, greater grapevine (*Vitis* spp., L.) presence, less shrubs, steeper slopes, and less white oak (*Quercus alba*, L.) abundance. With this new knowledge we can incorporate additional features of habitat in the formulation of a management plan for this declining species.

## Introduction

The least studied aspect of animal biology, which ornithologists usually do not explore, is the nocturnal life of birds [1]. Yet, this aspect of a birds’ ecology should be of interest to biologists. For example, the transmission of zoonotic diseases, such as West Nile Virus, has been linked to the roosting characteristics of American Robins (*Turdus migratorius*, L.) [2]. The few studies that have examined roosting ecology have concentrated on species that exhibit communal roosting behaviors such as Long-tailed Tits (*Aegithalos caudatus*, L.) and some corvid species such as Common Ravens (*Corvus corax*, L.) [3,4]. However, recent evidence suggests that some forest-interior species roost away from their diurnal home ranges [5].

Largely due to habitat loss on the breeding, wintering, and migratory stopover locations, migratory songbirds have been in decline for decades [6–12]. To obtain comprehensive knowledge of habitat requirements for a species, roosting habitats need to be considered [5]. In some instances, the habitat requirements for diurnal use areas and nocturnal (roosting) use areas may be similar, like that of wintering Ovenbirds (*Seiurus aurocapilla*, L.), which tend to roost in the core of their diurnal home range [13]. But, this may not be true of every species. Therefore, it is important to explore all aspects of a species’ ecology, especially in declining species. Roosting behavior on wintering grounds has been studied more often [i.e., 13–17] than on the breeding grounds [i.e., 5, 18, 19], but to get a full understanding of a species habitat needs, it is essential to study roost site preferences during the breeding season, and even on migratory stopover sites [i.e., 20].

Cerulean Warblers (*Setophaga cerulea*, Wilson) are one of the fastest declining species of wood-warbler in North America (~3% per yr) [12]. They are a small migratory songbird that breeds in hardwood forests of the eastern United States [21]. Throughout its range, they are listed as a species of conservation concern by the U.S. Fish and Wildlife Service [22], but in Indiana, Cerulean Warblers are an endangered species [23]. The International Union for Conservation of Nature (IUCN) classifies Cerulean Warblers as ‘near-threatened’ [24]. Habitat loss on both their breeding and wintering grounds has attributed to their rapid decline [22, 25]. Cerulean Warblers have been studied throughout their breeding range to some extent. However, due to their habits of nesting and foraging high in forest canopies, researching these small birds is quite a challenge, and therefore, a great deal about Cerulean Warblers is still unknown [26]. To date, there are no published quantitative studies of microhabitat characteristics at roost locations for Cerulean Warblers. The objectives of our study were to examine habitats associated with roost sites and to determine where roost sites were located, in relation to nests and territories. For the first time, we discuss microhabitats at Cerulean Warbler roost sites, explore roosting behavior, and offer forest management recommendations.

## Materials and methods

### Study area

**Fig 1:**
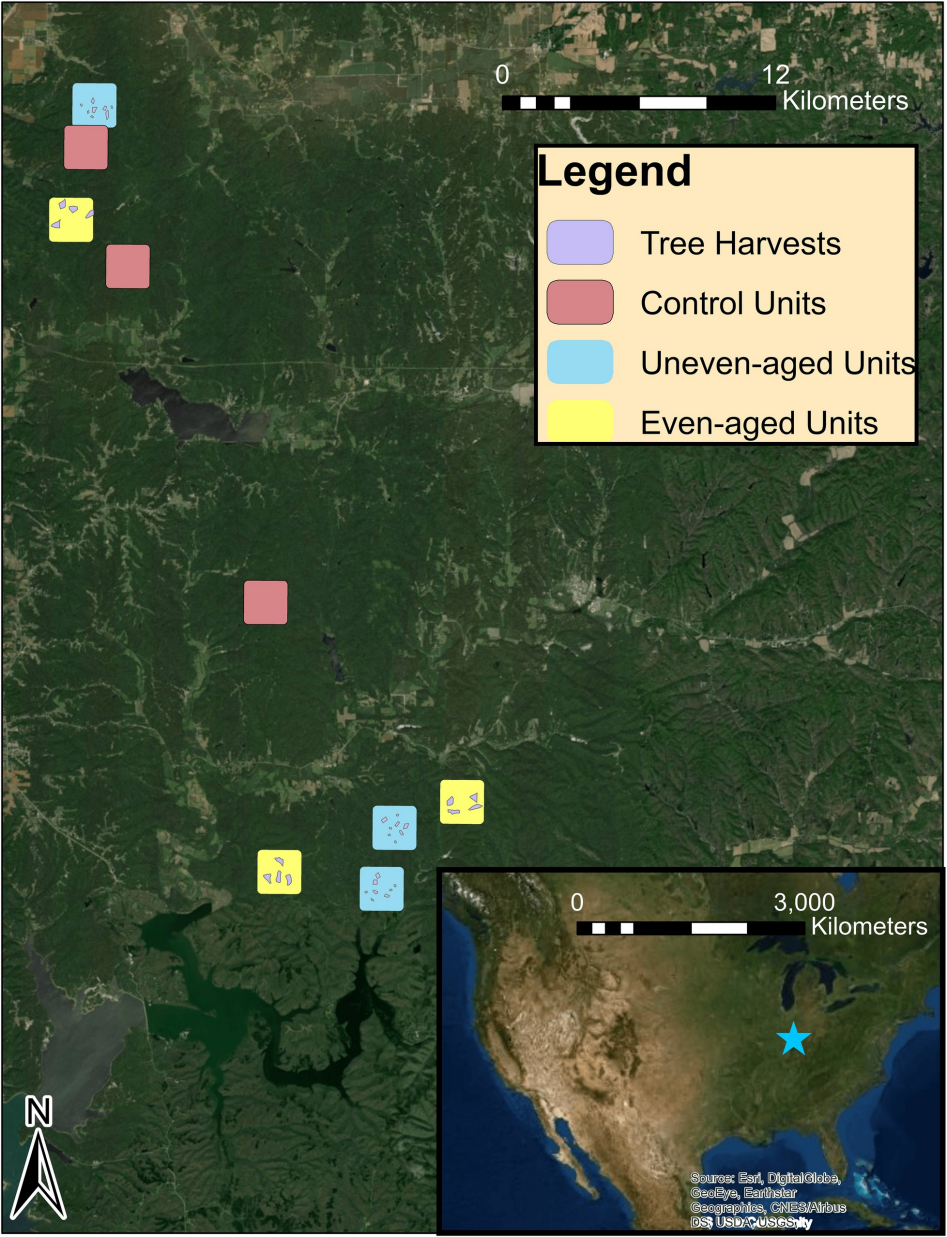
Hardwood ecosystem experiment study units. Research on roost habitat selection in Cerulean Warblers (*Setophaga cerulea*, Wilson) was conducted at 9 research sites in Morgan-Monroe and Yellowwood state forest in Morgan, Monroe, and Brown counties, southern Indiana during May-June, 2017. Three units are managed as even-aged forests, three managed as uneven-aged forests, and three units are control sites with no forest management practices.

This research was conducted in southern Indiana as part of the Hardwood Ecosystem Experiment (HEE), a 100 yr project that examines the effects of forest management on a variety of plant and animal species (39.114° N, -86.322° W). The HEE is located in Yellowwood and Morgan-Monroe state forests in Morgan, Monroe, and Brown counties [27]. These mature and second growth forests, located in the Highland Rim Natural Region in the Brown County Hills Section, are characterized by narrow ridgetops, steep slopes (20–40%), and hollows < 200 m wide, with elevation ranging from 150 to 290 m [28]. The forests are similar in plant species composition, historically dominated by oak-hickory species. The upper slopes of ridges are dominated by an almost pure stand of chestnut oak (*Quercus montana*, Willd.), and by a thick growth of greenbriar (*Smilax* spp., L.). In contrast, American beech (*Fagus grandifolia*, Ehrh.), red oak (*Quercus rubra*, L.), sugar maple (*Acer saccharum*, Marshall), and white ash (*Fraxinus americana*, L.) are common mesic species that dominate the ravines [29]. This region contains dissected uplands underlain by sandstone, siltstone, shale, and limestone with silt-loam soils [30]. Of 9 research sites (ranging in size from 303–483 ha), three research sites received even-aged management, three uneven-aged management, and three served as control sites with no forest manipulation (Fig 1) [27]. Even-aged forest management sites consist of two 4 ha clearcuts and two 4 ha shelterwood harvests, while uneven-aged forest management sites consist of eight patch-cuts consisting of four 0.4 ha, two 1.2 ha and two 2 ha size cuts, and a single tree selection throughout the remainder of the site [27].

### Territory demarcation

During May-June, 2017, territory demarcation was facilitated by following color-banded adult male Cerulean Warblers. Global Positioning System (GPS) points were taken and uploaded into a Geographic Information System (GIS) [31] at perches where Cerulean Warbler males sang and were tagged with flagging tape. Territories were visited multiple times so that sufficient territory points could be flagged.

### Nest searching and monitoring

We searched for nests throughout the breeding season. Territories from previous breeding seasons were revisited to search for returning birds. Once a nest was found, we monitored the nest every 1–3 days, depending on the stage of the nest (building, incubation, nestling); nests were monitored more frequently as the estimated date of fledging neared.

### Capture, banding, and auxiliary marker attachment

To capture male Cerulean Warblers for this study, we erected mist-nets on the ground and played conspecific songs or calls to lure birds into the net with an Altec Lansing H2O Bluetooth Waterproof speaker. Each captured male Cerulean Warbler was banded with an aluminum United States Geological Survey (USGS) leg band followed by 2–3 color bands that allowed us to identify individuals without recapture. Birds were aged as either second-year (SY) or after-second-year (ASY) according to wing molt characteristics [32]. Before release, a 0.33 g radio-transmitter (Blackburn Transmitters, Nacogdoches, TX, USA) was attached to each male Cerulean Warbler. We attached transmitters using the Rappole and Tipton [33] method, but with modifications discussed by Streby et al. [34]. Harnesses for transmitters were made of an elastic sewing thread that degrades and falls off after about 40 days [34].

To capture, band, and attach auxiliary markers on Cerulean Warblers, we obtained a state collecting permit through the Indiana Department of Natural Resources (IDNR) and a federal bird banding permit through the U.S. Geological Service (USGS; Permit #21781). Ball State Institutional Animal Care and Use Committee (IACUC) approval was also obtained to conduct this research (IACUC approval 437484–4).

### Tracking and observations

We tracked individuals starting 1 h after sunset to find their roost locations [5, 19]. We used a headlamp (Kohree KL11LM-10 hunting light, Charlotte, NC, USA) to visually locate roosting birds, and to identify the roost tree and height. Our objective was to attach a transmitter on each male associated with a nest. Due to early morning fieldwork, we only radio-tracked all adult male Cerulean Warblers to roost sites every Monday, Wednesday, and Friday from ~2200–0200 h to determine where individuals were roosting. Saturday and Sunday nights were used as alternate nights for tracking in case weather did not permit tracking on a given weeknight. Tracking was completed via honing in on the signal transmitted by the bird’s radio-transmitter. Once we were close to the signal, we circled around trees to identify the tree where the bird was likely roosting. We recorded the bird’s unique identification (alphabet given to separate from other individuals), date, time of observation, study unit, transmitter frequency, time of sunset, bird status (paired or not paired), nest status (building, laying, incubation, nestlings, or with fledglings), name of observers, GPS coordinates, roost tree species, and estimated roost height. To obtain roost height, we estimated the height from the ground to where the signal from the radio-transmitter was strongest in the tree.

### Microhabitat sampling

Throughout July 2017, vegetation data were collected at each roost tree and random location selected within the demarcated territory associated with each individual male. Random points were randomly generated within each bird’s respective territory using ArcGIS 10.3.1 [28]. The number of random points in each territory was equal to the number of nights that each bird was tracked to its roost site. Vegetation survey plots were 15 m in radius from the center point. A center point was directly under the roost site or at precise GPS coordinates for random points within territories. We recorded the date and point identification (bird, roost location, or random point name/number). Aspect was calculated using a compass to determine the direction of the slope (0–360). Slope was obtained using a clinometer at 11.3 m from the center point in the uphill and downhill directions; we used averages between the two measurements to calculate each plot’s slope. The presence or absence of canopy cover and ground cover from the center point were recorded from 0 to 10 m at 2 m intervals in each cardinal direction. A densitometer was used to identify canopy cover, while ground cover was determined by visually examining the ground where the survey flag entered the soil at every 2 m intervals [35–37]. Shrubs were counted and grouped into 2 categories (< 3 cm diameter at breast height [DBH] and 3–10 cm DBH); shrubs were only counted within a 5 m radius of the center point. Trees (any woody vegetation > 10 cm DBH) within 11.3 m radius of the center point, were also measured. In each quadrant of the survey plot, the tallest tree (m) was measured for height. The only measurement that was recorded at the 15 m radius from the center point was vertical vegetation stratification (density). We used a 2.5 m tall density board that was taped off into 5 sections; each section that was blocked by live vegetation was assigned a percent value of cover by the individual recording the data. We recorded presence/absence of grapevine within 11.3 m.

In addition to these measurements, one photograph of the canopy was taken at the center point of each roost location and each random point to include canopy density. Photographs were taken during the day using a Canon Rebel T5i camera with a fixed Canon EF 50 mm f/1.8 II lens. For standardization, the camera was placed 1.25 m above the ground on a levelled Vanguard ABEO 243AV tripod pointing north. The photographs were edited and transformed using ImageJ software [38]. Once a photograph was opened in ImageJ, we used the split channels function and selected for the blue channel to analyze canopy density, similar to methods described in Peterson et al. [39]. We turned the blue channel image into a binary image to characterize the canopy versus the sky. We opened the histogram of the binary image to reveal the number of pixels contained within the canopy. To determine canopy density, we divided the number of pixels within the canopy by the total number of pixels in the image.

### Statistical analyses

We calculated the number of shrubs at each point using the sum function in Excel, and then transferred the data onto the Comma-separated Values (CSV) spreadsheet. Similarly, we used various functions in Excel to calculate and transfer average canopy height (m), average vertical stratification (%), number of grapevine stems within 5 m, presence/absence of grapevine, average tree DBH height, number of trees, tree species that were favored by Cerulean Warblers for nesting (%), abundance of white oak trees (%; *Quercus alba*, L.), ground cover (%), canopy cover (%), canopy density (%), aspect using Beers’ aspect transformation [40], and averaged uphill and downhill slopes (%).

The statistical analyses were performed in program R [41]. Data and code are stored in an online repository located at https://figshare.com/s/62765784a5e2a437ea70. We began the analyses with a Spearman correlation test to identify auto-correlated variables. We chose a correlation coefficient of 0.60 as our ‘cut-off point’ to exclude variables that were auto-correlated [42, 43].

After reducing the number of parameters, we scaled the continuous variables in the dataset to z-scores. Next, we created a data frame that included each individual point, along with the type of point (roost or random), and any non-continuous variables. We used generalized-linear models with mixed-effects to account for non-independence among the samples (each bird tracked had multiple points). We created a model for each combination of variables and included the mixed-effect function into each model. After creating the models, we calculated summary statistics for each model to analyze Akaike Information Criterion (AIC) values. We transformed these values to second-order AIC (AICc) values to account for small sample sizes. We created a table of the AICc values and used only models with AICc values of < 2, which we considered to be equally plausible models. We used model averaging to identify which variables in our accepted models were most important. The model-averaged coefficients were used to make predictions on presence of roost sites based on every covariate within the selected models.

## Results

During May-June 2017, 10 adult male Cerulean Warblers were outfitted with radio-transmitters and tracked at night to their nocturnal roosting locations (Table 1). Nine males were aged as after-second-year individuals (ASY) and one was aged as a second-year individual (SY). The number of nights each bird was tracked ranged from 1–10 nights, depending on the life of the radio-transmitter. Nine adult males were known to be paired with a female based on diurnal observations; pairing status was unknown for one adult male Cerulean Warbler. Nest status was known for six of the adult male Cerulean Warblers that were tracked. We were unable to visually locate a Cerulean Warbler on its roost; however, based on the strongest radio signals from within the tree, Cerulean Warblers seemed to roost high in the canopy and within a denser area of vegetation. Estimated roost heights averaged 18.3 m (range 10–25 m). Territories were demarcated for all 10 adult males that were tracked to roost locations. The number of perch trees demarcated for each male territory ranged from 5–10. Of 50 identified roosts, 82% of roost sites were located outside of the bird’s demarcated territory. The average distance from the territory center to the roost site was 68.34 m (range: 3.81–267.7 m; n = 10), and of the birds with known nests, the average distance from the nest (while active) to the roost site was 85.21 m (range: 1.99–151.24 m; n = 6). Excluding active nest status, the average distance from roost site to nest was 91.49 m (1.99–264.7 m; n = 6).

**Table 1:** Cerulean Warbler roost site characteristics. Cerulean Warbler (*Setophaga cerulea*, Wilson) roost site statistics in southern Indiana, May-June 2017.

Bird	Age Class	# Roosts in Territory	# Roosts outside Territory	Min. distance to nest (m)	Max. distance to nest (m)	Min. distance between roosts (m)	Max. distance between roosts (m)	# Perch trees used to demarcate territory
A	ASY	0	6	26.1	95.3	14.2	107.2	7
B	ASY	1	9	-	-	6.7	241.2	5
C	ASY	1	7	2.0	142.6	13.6	140.6	7
D	ASY	0	1	-	-	-	-	10
E	SY	0	2	99.2	124.5	27.4	27.4	7
F	ASY	1	2	-	-	8.7	37.1	6
G	ASY	4	6	43.6	151.2	9.6	186.8	6
H	ASY	0	1	-	-	-	-	7
I	ASY	1	7	29.8	72.1	7.6	315.1	6
J	ASY	1	0	-	-	-	-	5
Total/average		9	41	40.14	117.14	12.54	150.77	6.6

Data missing in the 4 distance columns were a result of only one roost being recorded and/or nest location was unknown. Minimum and maximum distances apply to nest when they were active. In the case of Bird “I” roosting within its territory, this instance was after the nest had successfully fledged young; only the first two roost locations for this bird were while the nest was still active. ASY = After Second Year; SY = Second year.

Seventy-four percent of the roost trees selected by male Cerulean Warblers were also tree species favored for nesting (Table 2). White oak was the most common tree where Cerulean Warblers roosted (22%) followed by pignut hickory (*Carya glabra*, Miller) and sugar maple, each at 16%. Red oak and tuliptree (*Liriodendron tulipifera*, L.) each comprised 10% of roost trees. Roost trees of lesser importance were black oak (*Quercus velutina*, Lam.; 6%), big-tooth aspen (*Populus grandidentata*, Michaux; 4%), chestnut oak (2%), chinquapin oak (*Quercus muehlenbergii*, Engelm.; 2%), and sassafras (*Sassafras albidum*, Nees; 2%).

**Table 2:** Cerulean Warbler roost trees. Cerulean Warbler (*Setophaga cerulea*, Wilson) roost sites (n = 50) by tree species (%).

Roost Tree Species	# of Trees	% of Roost Trees
**White Oak (*Quercus alba*, L.)**	**11**	**22**
**Pignut hickory (*Carya glabra*, Miller)**	**8**	**16**
**Sugar maple (*Acer saccharum*, Marshall)**	**8**	**16**
Red oak (*Quercus rubrum*, L.)	5	10
**Tuliptree (*Liriodendron tulipifera*, L.)**	**5**	**10**
**Black oak (*Quercus velutina*, Lam.)**	**3**	**6**
Big-tooth aspen (*Populus grandidentata*, Michaux)	2	4
**Chestnut oak (*Quercus montana*, Willd.)**	**1**	**2**
Chinquapin oak (*Quercus muehlenbergii*, Engelm.)	1	2
**Sassafras (*Sassafras albidum*, Nees)**	**1**	**2**
Unknown trees	5	10

Roost Tree Species in bold represent tree species used by Cerulean Warblers for nesting. If a specific roost tree could not be identified due to multiple tree canopies coming into contact, it was placed into the unknown category.

The number of variables used in model building were reduced from 15 to 9 to reduce multicollinearity. Six models were selected based on ΔAICc scores of < 2.0 (Table 3). Variables included in the top 6 models were number of shrubs, percent white oak, percent canopy cover, slope, basal area, and presence of grapevine. Percent white oak and number of shrubs were the most important variables and appeared in all 6 models.

**Table 3:** Top models for presence of Cerulean Warbler roost sites. Top 6 models selected for presence of roost locations of Cerulean Warblers (*Setophaga cerulea*, Wilson) in southern Indiana in May-June 2017, with AICc values, degrees of freedom, and weights.

Models	ΔAICc	k	Weight
Shrubs + white oak + canopy cover	0	5	0.0661
Shrubs + white oak + canopy cover + slope	0.4	6	0.0492
Shrubs + basal area + white oak + canopy cover	0.9	6	0.0328
Shrubs + basal area + white oak +canopy cover + slope	1.1	7	0.0303
Shrubs + white oak	1.6	4	0.0256
Shrubs + grapevine + white oak + canopy cover	1.9	6	0.0235

**Fig 2:**
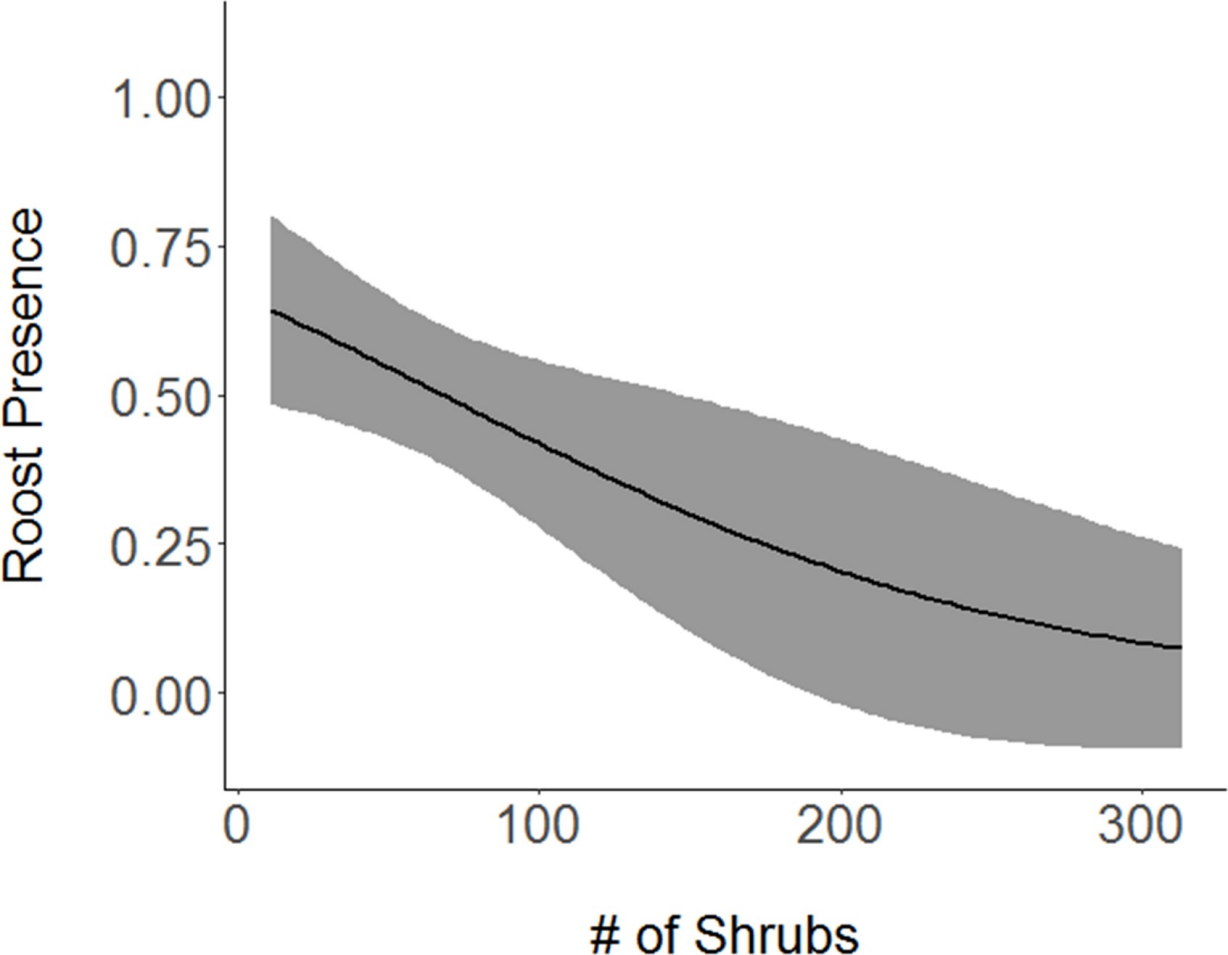
Association between Cerulean Warbler roosts and shrubs. Cerulean Warbler (*Setophaga cerulea*, Wilson) roost sites were negatively associated with an increased number of shrubs in Morgan-Monroe and Yellowwood state forests, southern Indiana, 2017. The gray area represents 95% confidence intervals. Original data are shown on the x-axis.

**Fig 3:**
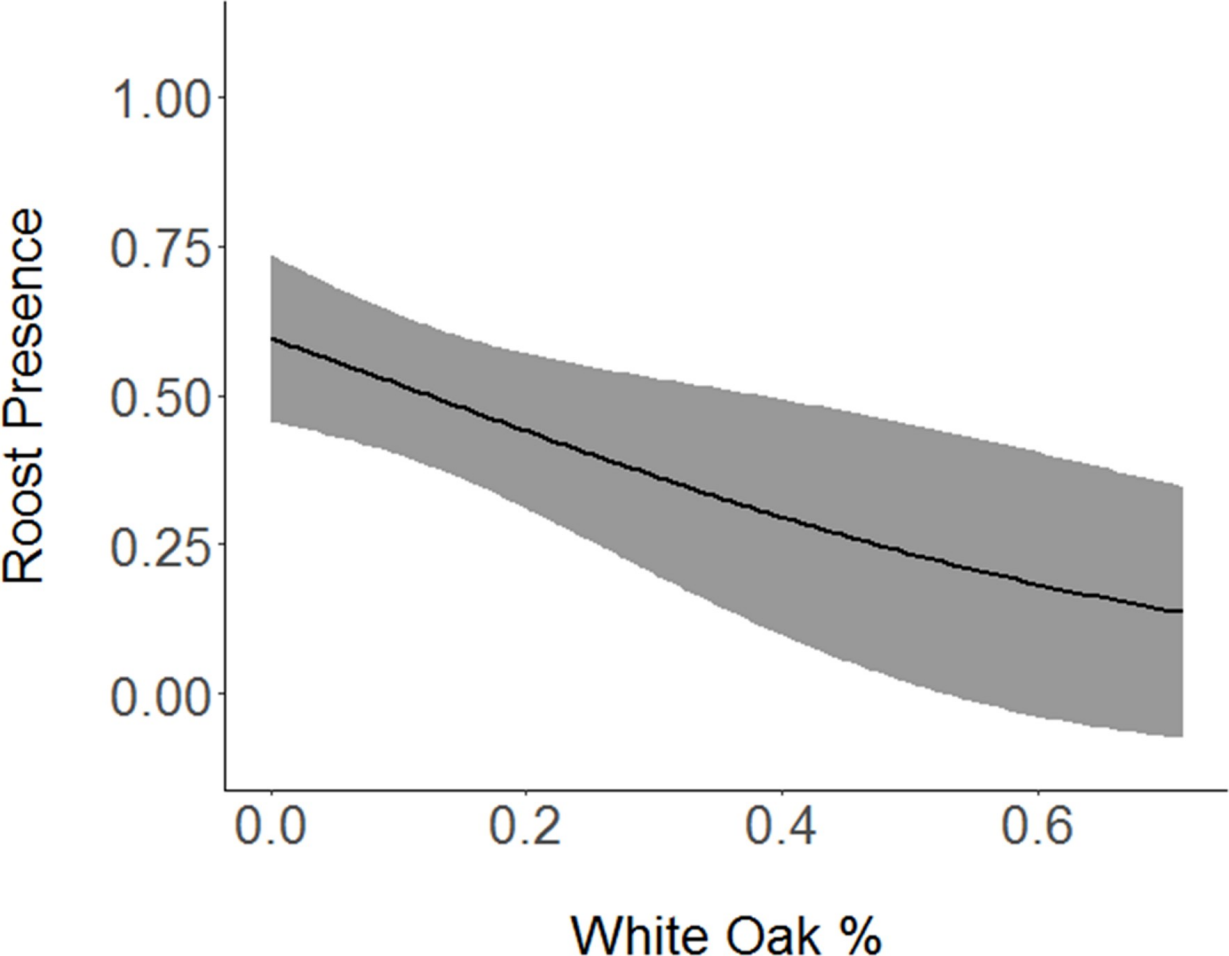
Association between Cerulean Warbler roosts and white oak. Cerulean Warbler (*Setophaga cerulea*, Wilson) roost sites were negatively associated with increased white oak (*Quercus alba*) abundance at Morgan-Monroe and Yellowwood state forests in southern Indiana, 2017. The gray area represents 95% confidence intervals. Original data are on the x-axis.

**Fig 4:**
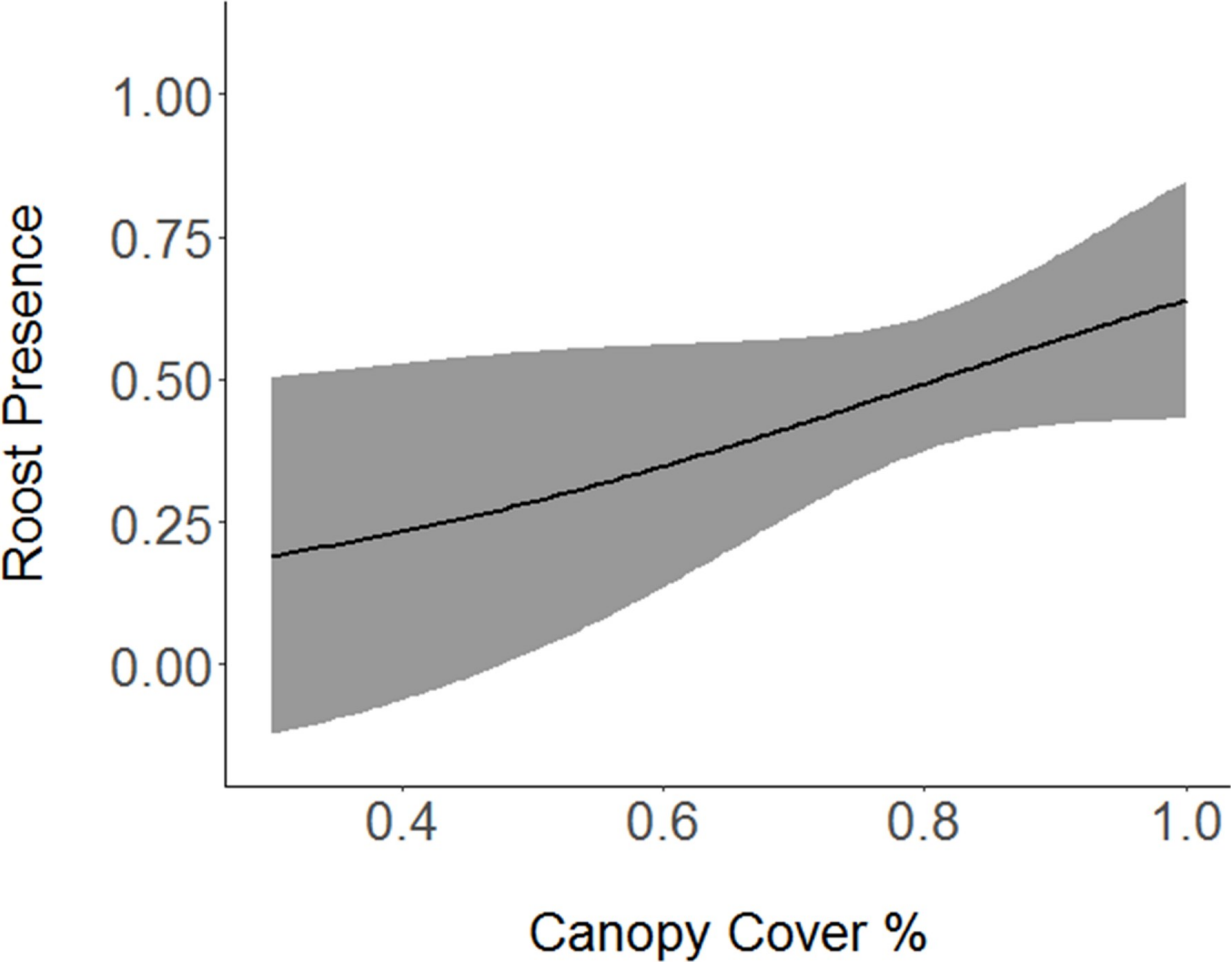
Association between Cerulean Warbler roosts and canopy cover. Cerulean Warbler (*Setophaga cerulea*, Wilson) roost sites were positively correlated with greater canopy cover at Morgan-Monroe and Yellowwood state forests in southern Indiana, 2017. The gray area represents 95% confidence intervals. Original data are on the x-axis.

Shrubs, with a model averaged coefficient of -0.68104 (Table 4), had a strong negative association with roost sites (Fig 2). An equally important variable, the percentage of white oak in each vegetation survey, also had a strongly negative relationship (-0.58777) with presence of roost sites (Fig 3). Percent canopy cover had a strong positive relationship (0.47470) with roost presence (Fig 4). Slope had a slightly positive association (0.12082) with roost presence, and basal area, the least important variable in the selected models, had a slightly negative relationship (-0.08452) with roost preference. Grapevine showed a slight positive relationship (0.0.03372) with the presence of roost sites.

**Table 4:** Model-averaged coefficients of Cerulean Warbler roost sites. Model-averaged coefficients (full average) for Cerulean Warbler (*Setophaga cerulea*, Wilson) roosts compared with random points in southern Indiana, May-June 2017 with model-averaged coefficients (full average).

	Estimate	Std. Error	Adjusted SE	Z value	Pr(>|z|)	Importance	N models
Intercept	-0.07346	0.25075	0.25396	0.289	0.7724	-	-
Shrubs	-0.68104	0.32078	0.32484	2.097	0.0360	1.00	6
White oak	-0.58777	0.28142	0.28505	2.062	0.0392	1.00	6
Canopy cover	0.47470	0.31984	0.32267	1.471	0.1412	0.88	5
Slope	0.12082	0.24978	0.25152	0.480	0.6310	0.33	2
Basal area	-0.08452	0.18354	0.18478	0.457	0.6474	0.30	2
Grapevine	0.03372	0.20050	0.20248	0.167	0.8677	0.10	1

## Discussion and conclusion

**Fig 5:**
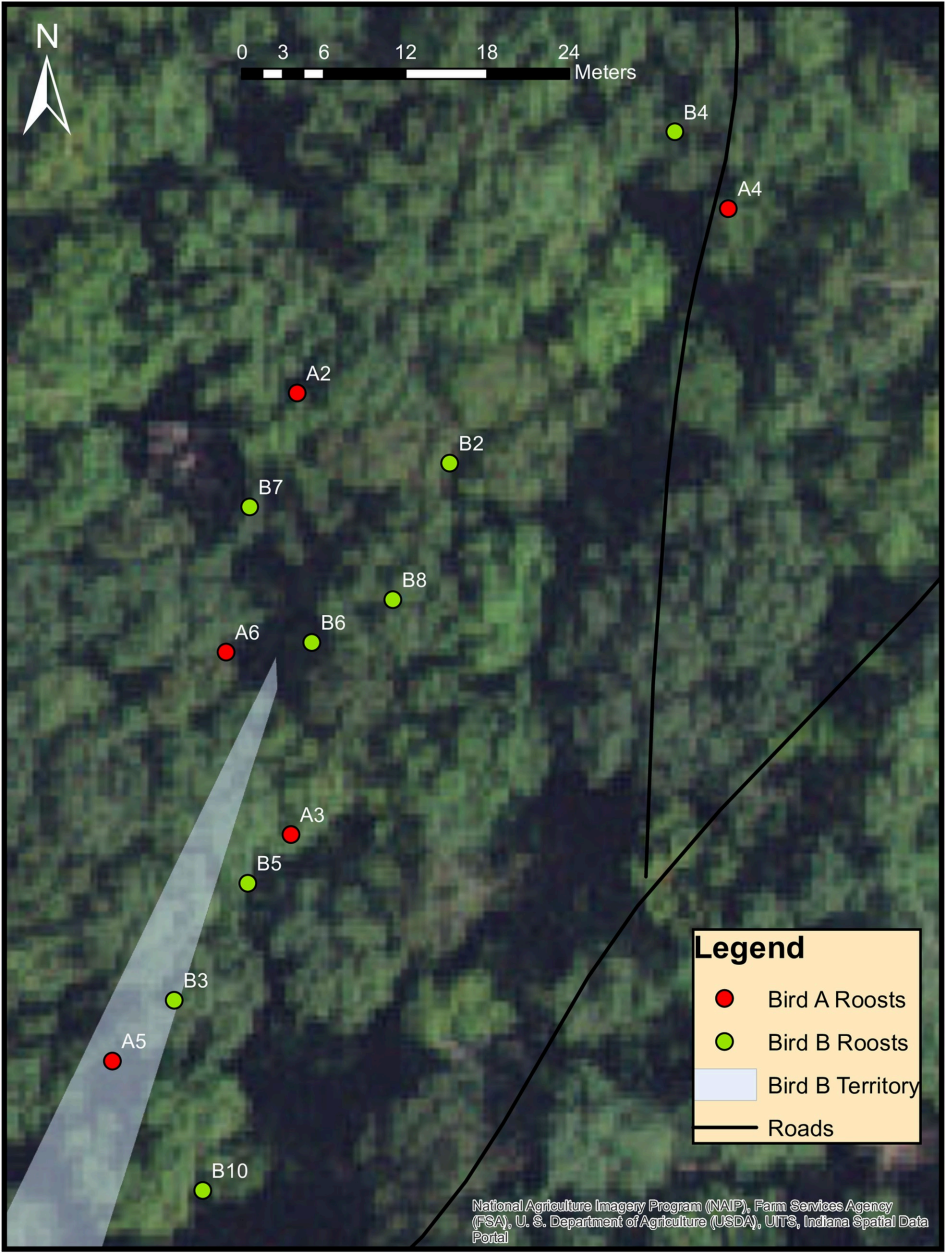
Neighboring male Cerulean Warbler roost locations. Locations of Cerulean Warbler (*Setophaga cerulea*, Wilson) “A” who roosted near Cerulean Warbler “B” across multiple nights. Bird “A” was never found to roost within his demarcated territory. The numbers shown next to roost points correspond to the day of tracking. Bird “A” had a failed first nest attempt followed by a successful second attempt; the nest for bird “B” was never found. Both birds were tagged on the same day; thus, the days of tracking are the same for each individual. The closest these individuals came to roosting near each other was 6 m on night 6 of tracking. This figure was produced with a scale less than 1:1000, therefore, the satellite imagery basemap appears pixelated.

In general, most male Cerulean Warbler roost sites were not near nests. Many roost sites were located outside of defended territories, and in fact, some birds roosted near each other or in the direction of neighboring territories. In some cases, we noticed a few interesting relationships when plotting roost points for individual birds in ArcGIS. For instance, one bird was never found roosting in its territory, but was found roosting in the territory of a neighboring male Cerulean Warbler. On most of the nights that this bird was tracked to a roost site, it was found roosting near a neighboring Cerulean Warbler that was also tagged with a radio-transmitter (Fig 5, S1 Fig). Another bird that defended a territory near the top of a slope was always found roosting to the west of its nest in the direction of another neighboring male (S2 Fig). One male Cerulean Warbler that nested in a shelterwood harvest in one of the even-aged stands chose to roost outside of its territory at the top of the shelterwood cut near a logging road (S3 Fig). One male Cerulean Warbler appeared to favor roosting at the edge of conifer stands near its territory (S4 Fig). Once its nestlings fledged, this bird moved to a number of different roost sites, likely due to its fledglings moving further from the nest tree each day (S1 Table), which is common among Cerulean Warblers [34]. The roost sites of the fledglings with respect to the adult were unknown. At a control site, a Cerulean Warbler roosted inside its territory once, which was very close to the nest site, but at other times this bird was found roosting outside of his territory in different directions (S5 Fig). This bird was believed to have a second nest with a different female Cerulean Warbler on the opposite side of the road, but that nest was never found. This bird’s territory was located in the buffer zone of a control site, which was subjected to light harvesting.

The presence of grapevine, white oak, and canopy gaps are often selected by Cerulean Warblers during territory establishment and nest site selection [44]. While many of the variables at roost sites were similar to variables at nest sites within territories, there were some differences. For instance, white oak abundance was much lower near roost sites compared to territories where birds nest. Canopy cover was also higher at roost sites than in territories. One similarity between territory and roost sites was the presence of a greater abundance of grapevine.

Tree species used for roosting varied, but roost sites were most commonly found in white oak (22%). Cerulean Warblers do not use red oak as a nesting tree in our study sites, yet 10% of roost sites were located in this species. Typically, Cerulean Warblers were found roosting in tree species that they use for nesting (74% of roost trees). Adult male Cerulean Warblers selected roosts that had increased amounts of canopy cover and less shrubs, which suggests a closed canopy. Small birds lose heat at night, even in warm climates [45], and denser vegetation may help insulate the birds and allow them to maintain a constant body temperature. In addition, increased vegetation density at roost sites may also play a role in predator avoidance. Higher vegetation densities were suggested as a predator avoidance strategy for the Sichuan Partridge (*Arborophilia rufipectus*, Boulton) [46]. Jirinec et al. [5] also found that Wood Thrush (*Hylocichla mustelina*, Gmelin) roost sites had increased vegetation density. We found a slightly negative relationship with basal area at roost sites, which suggests that the stand density was less at roost sites than within random points. In addition, fewer shrubs and higher canopy density at roost sites suggests Cerulean Warbler roosts were likely in trees with full, dense crowns. Although the presence of grapevine was the least important covariate (importance factor = 0.10), it was found to have a positive relationship with roost sites. Grapevine bark is the primary source of nest building material for Cerulean Warblers. Grapevine can also create a dense canopy when it reaches the tops of trees and could be favored as a place to roost within a roost tree.

Even though white oak was the most selected roost tree, there was significantly less white oak surrounding roost sites compared to random points in each male’s territory. Cerulean Warblers favor white oak for nesting and foraging [35, 36, 47–49], but appeared to strongly prefer roost sites surrounded by fewer white oak. Many Cerulean Warbler territories were established in clusters of white oak trees at our study sites, which was a preferred nesting species. Cerulean Warblers exhibit clustered territoriality [50]; therefore, many territories are likely established around these clusters of white oak. With 82% of roost sites outside of territories and the preference to roost in white oak, Cerulean Warblers may actively seek white oak in areas where they are not concentrated.

Male Cerulean Warbler territories at our study sites were characterized by greater shrub cover [51]. In contrast, roost sites were found to have fewer shrubs. Cerulean Warblers prefer to place nests in white oak near canopy gaps. Areas such as these would have less canopy cover than roost sites, and more shrubs due to increased light to the forest floor. In a separate study at these sites, nest tree sites and territories were found to have higher densities of canopy cover than random sites. Territories were also characterized with steeper slopes than random sites [51]. The random points in our study were located within each bird’s territory, whereas Nemes and Islam [51] compared centralized territory points with random points outside of territories. Cerulean Warblers may have chosen to roost outside of their territories more often because they preferred to roost in areas with fewer shrubs and more of a closed canopy.

Jirinec et al. [5] found that SY (n = 16) male Wood Thrush commuted further to nocturnal roost sites than ASY (n = 18) individuals. In our study, most of the Cerulean Warblers that were banded were ASY birds. Only 1 out of 10 birds for this study was an SY individual; therefore, any comparison between age class was not possible for this study. Though, this individual never roosted as close to its nest as the other ASY individuals (Table 1)

Male Cerulean Warblers were never found to use the same roost; though a few roosts were found within 12 m of a previous roost. It may be more beneficial for birds to use different roosts each night, possibly to avoid predators. Cerulean Warblers are known to be colonial breeders with multiple territories neighboring one another [50]. In several instances, neighboring male Cerulean Warblers were found roosting near each other, sometimes within 8 m. Because Cerulean Warblers are very territorial [44], it is possible that these birds counter-sang with each other before sunset and decided to find a safe roost site until morning, at which time they would resume counter-singing and territorial defenses. Jirinec et al. [5] hypothesized that adult male Wood Thrush roost further away from their nest to solicit extra-pair copulations in the morning. In many birds, copulation occurs in the mornings (54%), or both during mornings and evenings (25%) [52]. In Wood Thrush, extra pair paternity has been documented in about 40% of the nestlings [53]. Few data exist on extra-pair copulations in Cerulean Warblers, but some data suggests that Cerulean Warblers participate in extra-pair copulations [54]. Extra-pair copulations were observed in banded Cerulean Warblers in eastern Ontario. Through blood samples, the researchers found that 57.1% of young (n = 7) were sired by a different male than the male Cerulean Warbler in the territory [54]. Male Cerulean Warblers may attempt to obtain extra-pair copulations with neighboring females when roosting further from their nests or outside of their territories.

Of the 50 Cerulean Warbler roost sites located, 82% of roost sites were found outside of the bird’s demarcated territory (18% of roosts found within the territory). In contrast, Carpenter and Wang [19] found that 36.6% of male Cerulean Warbler roosts were outside of their diurnal home ranges, and 13.6% were in core diurnal use areas. However, Carpenter and Wang [19] used kernel density estimators (KDE) and diurnal home ranges, which differ from territories. Diurnal use areas (home ranges) encompasses more area than a territory [55], which would suggest why we observed more roosts outside of territories. Although different, demarcated territories would likely be similar in size and function to core areas from the KDE [56, 57]. We found 18% of roost sites within territory boundaries, while Carpenter and Wang [19] found 13.6% of roost sites within core diurnal use areas. Similarly, we found 82% of roosts outside of territories, whereas Carpenter and Wang [19] found 86.4% of roost sites outside of core diurnal use areas.

Of the 50 roost sites located, we found one male Cerulean Warbler roost within the territory of a neighboring male Cerulean Warbler. In one instance, a male Cerulean Warbler in Alabama roosted inside another male’s core diurnal use area after traveling 2.2 km from its own core area [19]. Carpenter and Wang [19] also observed a case where a male Cerulean Warbler was found roosting near its active nest, though, they did not examine any influences of nest status, nor did they actively search for Cerulean Warbler nests in their study. We also note one instance where a male Cerulean Warbler roosted near its active nest, about 2 m away. Throughout Carpenter and Wang’s [19] study, only one male Cerulean Warbler was found to roost in the same tree more than once, whereas we did not observe any of our bird’s roosting in the same trees.

Carpenter and Wang [19] found that the average distance from roost sites to the center of the core area was 159 m (46–414 m; n = 9). In our study, 7 of 10 male Cerulean Warblers roosted within the same study unit where we estimated there were about 35 breeding pairs. The area where Carpenter and Wang [19] completed their study was estimated to contain 20 breeding pairs. The smaller average distances from territory center to roost site that we observed (68.34 m [3.81–267.7 m]) may be due to higher densities of Cerulean Warblers in our study area. Cerulean Warblers may not need to travel as far to get to neighboring male territories in our site to defend territories or to engage in extra-pair copulations.

Cerulean Warblers are thought to hold ‘all-purpose territories’ [44]. We found that 82% of roost sites were located outside of territories, while Carpenter and Wang [19] found that 86.4% of roost sites were outside of core areas, which are similar to territories [56, 57]. Ideally, more marked trees would have provided a higher likelihood of completely mapping a bird’s territory. Some of the 82% of roosts that were considered to be outside of each bird’s respective territories may actually have been inside the territory. However, all of our birds had territories that were satisfactorily mapped with five or more trees, and yet, still roosted outside of their territories. These results suggest that Cerulean Warblers do not hold ‘all-purpose territories’ that include roost locations. In multiple instances, male Cerulean Warblers were found to be outside of demarcated territories during the nesting period. One explanation for this observation may be that some male Cerulean Warblers were seeking extra-pair copulations with neighboring individuals.

### Management implications

In addition to incorporating aspects such as nesting and fledgling habitat preferences in any management plan for Cerulean Warblers to create the highest quality habitat, roost sites must also be considered. In our study sites, roost sites were characterized by greater canopy density with grapevine but with fewer shrubs and less basal area. Cerulean Warbler habitat use during the breeding season (nest building, nest tree selection, roost site selection [this study], fledgling dispersal, etc.) is heavily characterized by the presence of grapevines. Although the presence of grapevines can reduce timber quality [58], it should not be reduced within a stand. Cerulean Warblers preferred to roost in white oak more often than other tree species. More than 80% of nests in our sites were placed in white oak, which makes this species a very important tree for nesting. Clusters of Cerulean Warbler territories are often placed around clusters of white oak stands, and with birds roosting in white oak outside of territories, suggests that there is a preference for white oak across the landscape. With oaks in decline [59], focus should be on oak regeneration, in particular, white oak regeneration. It is easier to grow a hardwood forest than it is to grow a mixed-oak forest stand because more shade-tolerant species, such as American beech and sugar maple, outcompete oak trees for canopy dominance. Historically, forests in Indiana have been managed as uneven-aged stands where small patch-cuts are created as well as, single-tree removal. Additional research into roosting differences between age classes and sexes may prove useful to conserve habitats used by Cerulean Warblers. In addition, more data from other areas throughout their breeding and non-breeding distribution would be of great importance to determine if there is any geographic variation among roost site preferences in Cerulean Warblers.

## Supporting information

S1 Fig Roost sites of Birds A, B and J. Birds A and B were neighboring male Cerulean Warblers (*Setophaga cerulea*, Wilson) found roosting near each other on multiple occasions in Yellowwood State Forest, Indiana, USA, during May to June, 2017. Bird J was only located roosting for one night, after which, we believe the radio transmitter to have malfunctioned.
(TIF)

S2 Fig Roost sites of Birds F and G. Birds F and G were neighboring male Cerulean Warblers (*Setophaga cerulea*, Wilson) that roosted more upslope and west of their territories in Yellowwood State Forest, Indiana, USA, during May to June, 2017.
(TIF)

S3 Fig Roost sites of Bird E. Bird E was a Cerulean Warbler (*Setophaga cerulea*, Wilson) that nested in a shelterwood cut, and roosted near the top of the slope in Yellowwood State Forest, Indiana, USA, during May to June, 2017.
(TIF)

S4 Fig Roost sites of Bird H and I. Bird I was a Cerulean Warbler (*Setophaga cerulea*, Wilson) that nested in an even-aged study unit, and was found to move longer distances after its young successfully fledged in Yellowwood State Forest, Indiana, USA, during May to June, 2017.
(TIF)

S5 Fig Roost sites of bird C and D. Bird C was a Cerulean Warbler (*Setophaga cerulea*, Wilson) that nested at the edge of a control unit. This bird only roosted within its territory one night, while roosting outside of its territory during all other observations. Bird D was only tracked one night before the transmitter failed.
(TIF)

S1 Table Roost movements of Birds A, C, E, G and I. This table shows the distances from territory centers and distances from nests, along with corresponding nesting status of Cerulean Warblers (*Setophaga cerulea*, Wilson) in Yellowwood State Forest, Indiana, USA, during May to June 2017. Birds B, D, F and H are excluded because nests were not found for those individuals.
(DOCX)
