# Supplementary figures and images for: Analysis of microhabitat characteristics at roost sites of Cerulean Warblers
Source: PLoS One. 2020 Nov 3;15(11):e0241501. doi: 10.1371/journal.pone.0241501 (PMC7608899; doi:10.1371/journal.pone.0241501)

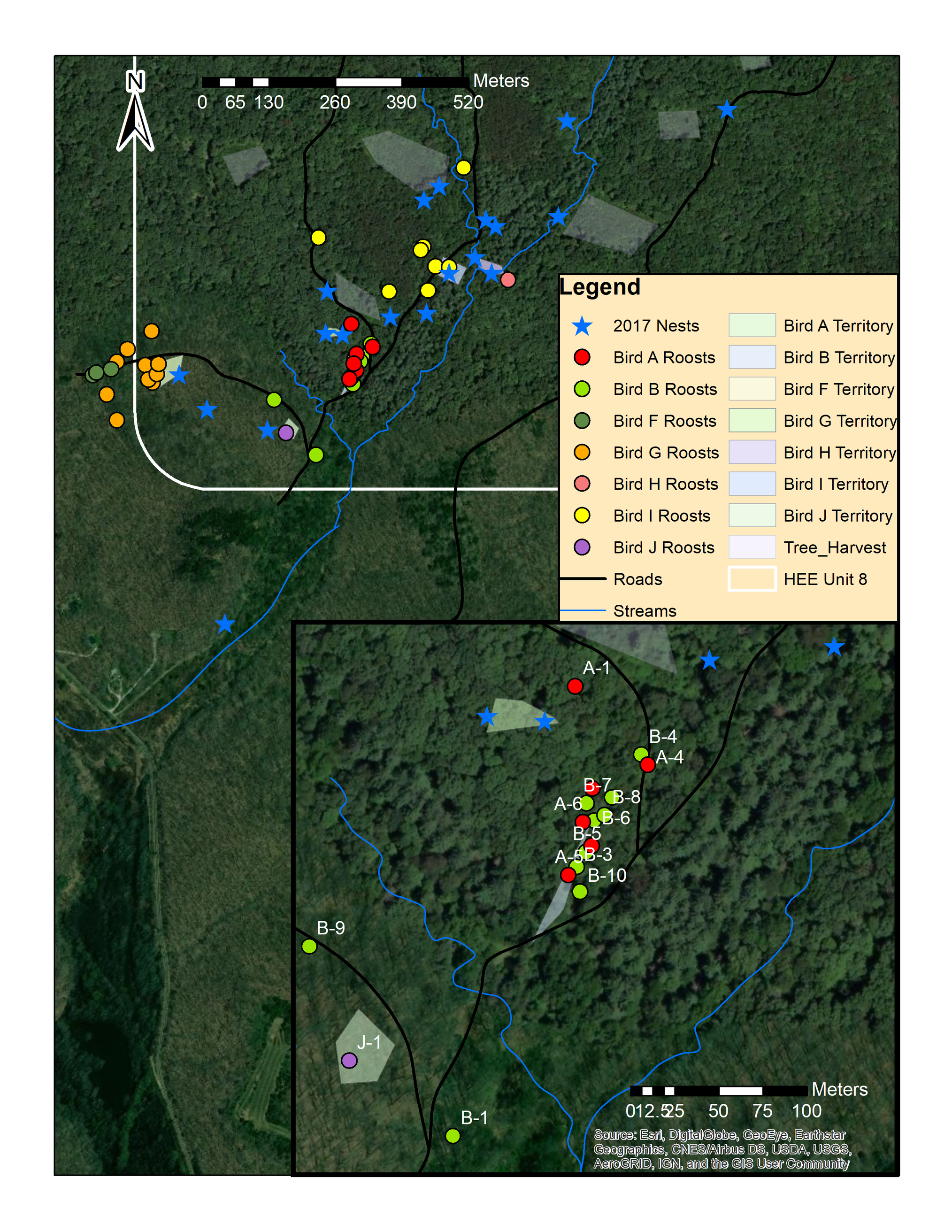

Supplement: S1 Fig — Roost sites of Birds A, B and J. Birds A and B were neighboring male Cerulean Warblers (Setophaga cerulea, Wilson) found roosting near each other on multiple occasions in Yellowwood State Forest, Indiana, USA, during May to June, 2017. Bird J was only located roosting for one night, after which, we believe the radio transmitter to have malfunctioned. (TIF) [file pone.0241501.s001.tif]

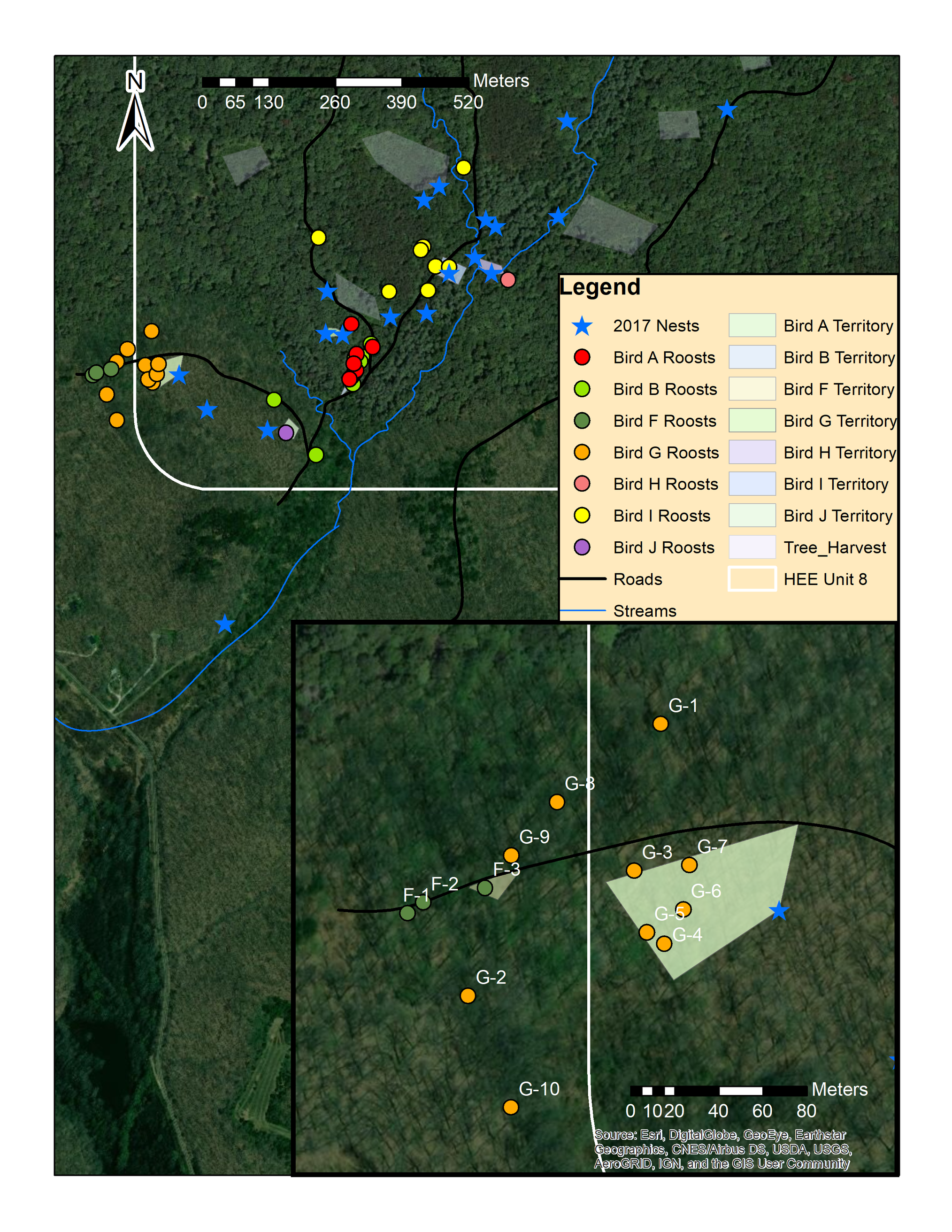

Supplement: S2 Fig — Roost sites of Birds F and G. Birds F and G were neighboring male Cerulean Warblers (Setophaga cerulea, Wilson) that roosted more upslope and west of their territories in Yellowwood State Forest, Indiana, USA, during May to June, 2017. (TIF) [file pone.0241501.s002.tif]

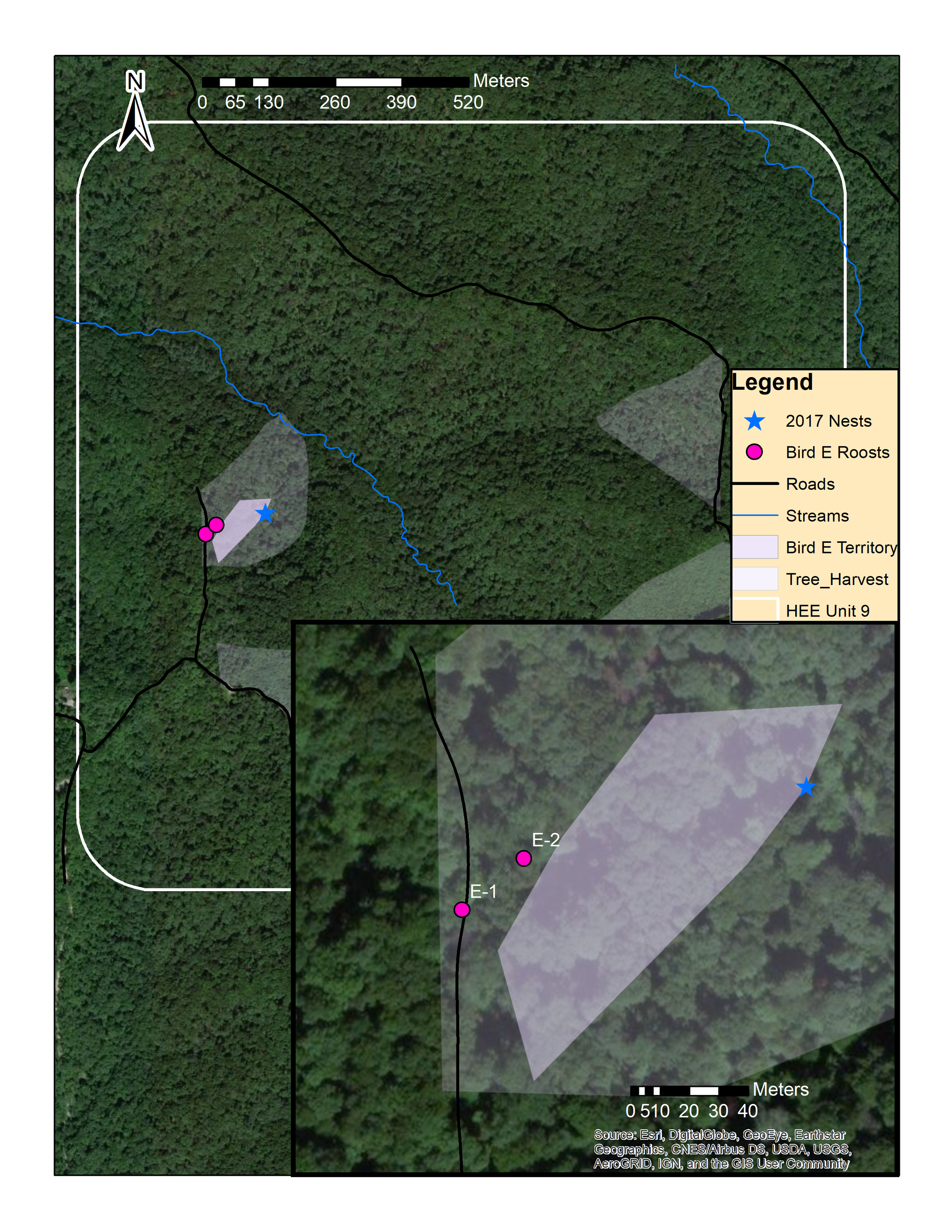

Supplement: S3 Fig — Roost sites of Bird E. Bird E was a Cerulean Warbler (Setophaga cerulea, Wilson) that nested in a shelterwood cut, and roosted near the top of the slope in Yellowwood State Forest, Indiana, USA, during May to June, 2017. (TIF) [file pone.0241501.s003.tif]

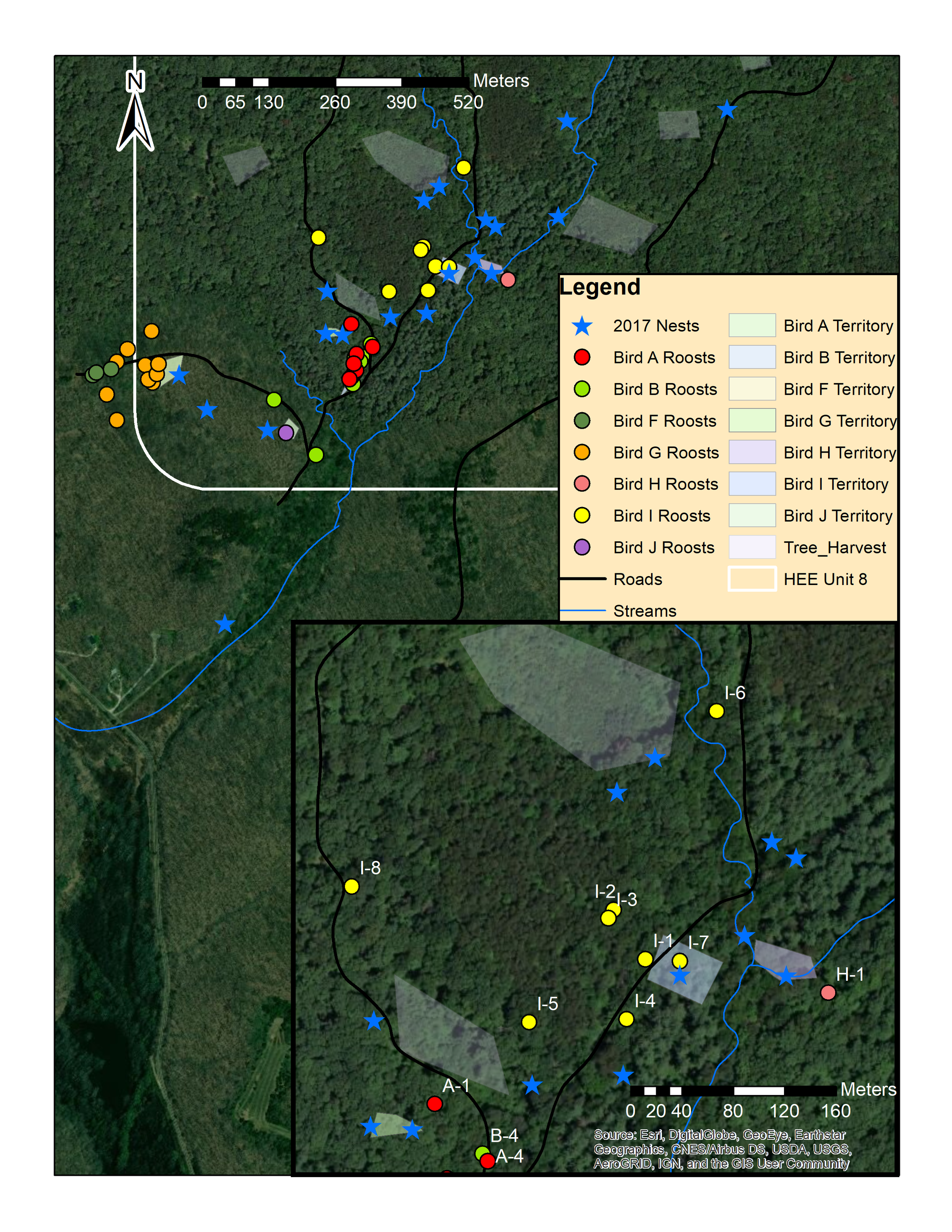

Supplement: S4 Fig — Roost sites of Bird H and I. Bird I was a Cerulean Warbler (Setophaga cerulea, Wilson) that nested in an even-aged study unit, and was found to move longer distances after its young successfully fledged in Yellowwood State Forest, Indiana, USA, during May to June, 2017. (TIF) [file pone.0241501.s004.tif]

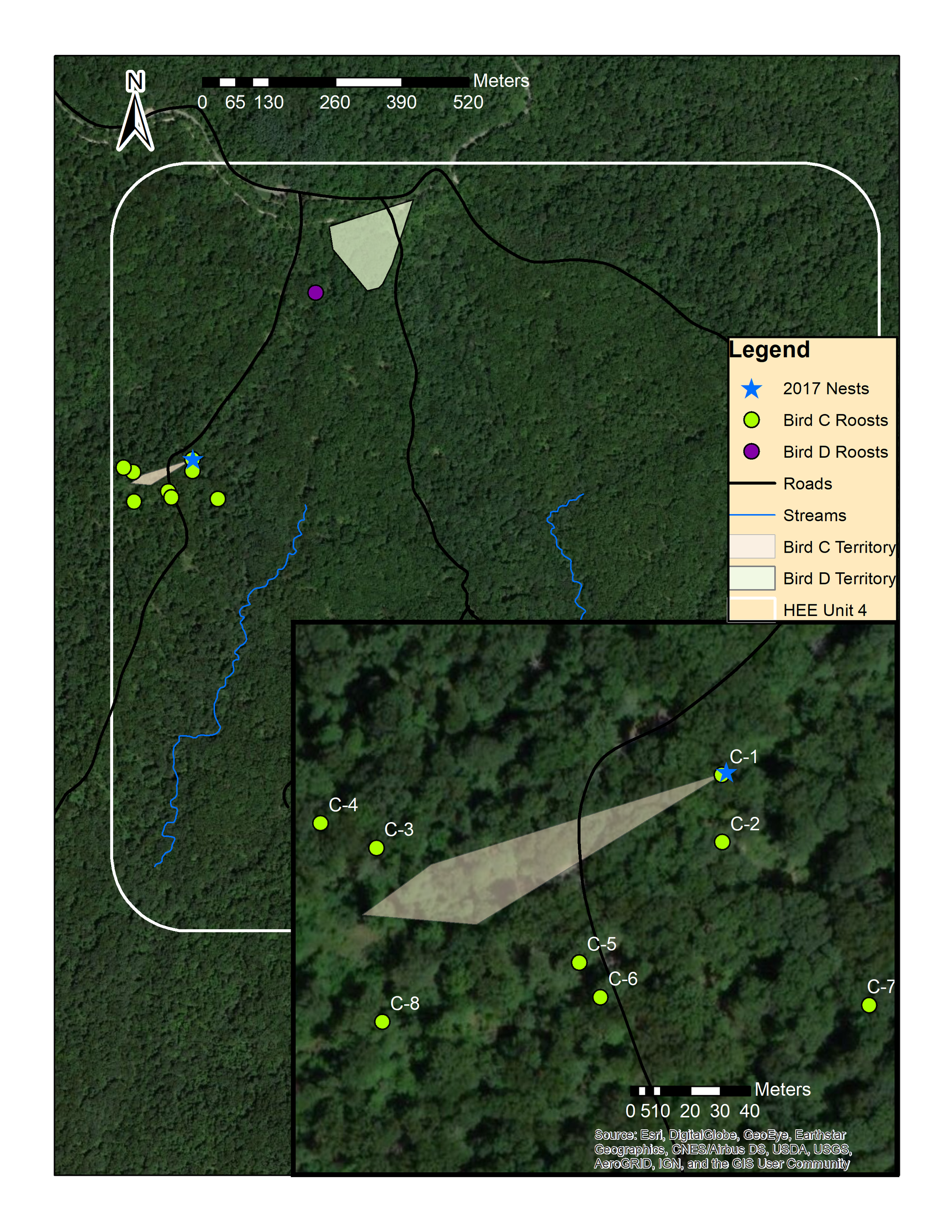

Supplement: S5 Fig — Roost sites of bird C and D. Bird C was a Cerulean Warbler (Setophaga cerulea, Wilson) that nested at the edge of a control unit. This bird only roosted within its territory one night, while roosting outside of its territory during all other observations. Bird D was only tracked one night before the transmitter failed. (TIF) [file pone.0241501.s005.tif]
