# Supplementary material for: Analysis of microhabitat characteristics at roost sites of Cerulean Warblers
Source: PLoS One. 2020 Nov 3;15(11):e0241501. doi: 10.1371/journal.pone.0241501 (PMC7608899; doi:10.1371/journal.pone.0241501)
Supplement: S1 Table — Roost movements of Birds A, C, E, G and I. This table shows the distances from territory centers and distances from nests, along with corresponding nesting status of Cerulean Warblers (Setophaga cerulea, Wilson) in Yellowwood State Forest, Indiana, USA, during May to June 2017. Birds B, D, F and H are excluded because nests were not found for those individuals. (DOCX) [file pone.0241501.s006.docx]

**S1 Table.**

| Bird ID | Roost # | Distance from territory center (m) | Distance from nest (m) | Nest status |
| --- | --- | --- | --- | --- |
| Bird A | 1 | 37.61 | 52.64 | Building |
|  | 2 | 59.82 | 72.39 | Failed (then re-nested) |
|  | 3 | 85.21 | 94.37 | laying |
|  | 4 | 79.93 | 95.26 | laying |
|  | 5 | 95.04 | 101.26 | Incubating |
|  | 6 | 71.29 | 81.07 | Incubating |
| Bird C | 1 | 74.76 | 1.99 | Building |
|  | 2 | 69.11 | 23.58 | laying |
|  | 3 | 47.57 | 120.18 | Incubating |
|  | 4 | 67.36 | 137.2 | Incubating |
|  | 5 | 38.9 | 80.79 | Incubating |
|  | 6 | 52.53 | 86.55 | Incubating |
|  | 7 | 127.1 | 91.64 | Nestlings |
|  | 8 | 69.49 | 142.62 | Nestlings |
| Bird E | 1 | 61.72 | 124.5 | Nestlings |
|  | 2 | 43.24 | 99.25 | Nestlings |
| Bird G | 1 | 81.63 | 100.13 | Incubating |
|  | 2 | 117.31 | 146.54 | Incubating |
|  | 3 | 35.39 | 68.3 | Incubating |
|  | 4 | 28.65 | 54.44 | Incubating |
|  | 5 | 31.61 | 61.03 | Nestlings |
|  | 6 | 12.16 | 43.67 | Nestlings |
|  | 7 | 16.88 | 45.66 | Nestlings |
|  | 8 | 80.84 | 112.18 | Nestlings |
|  | 9 | 90.98 | 124.15 | Nestlings |
|  | 10 | 130.35 | 151.24 | Nestlings |
| Bird I | 1 | 32.41 | 29.77 | Nestlings |
|  | 2 | 73.06 | 72.1 | Nestlings |
|  | 3 | 72.52 | 71.07 | Fledglings |
|  | 4 | 59.17 | 54.45 | Fledglings |
|  | 5 | 127.97 | 123.47 | Fledglings |
|  | 6 | 203.77 | 207.03 | Fledglings |
|  | 7 | 8.63 | 10.43 | Fledglings |
|  | 8 | 267.7 | 264.7 | Fledglings |
